# Long-Term Investment for Infants: Keys to a Successful Early Infant Male Circumcision Program for HIV Prevention and Overall Child Health

**DOI:** 10.9745/GHSP-D-15-00229

**Published:** 2016-07-02

**Authors:** Tin Tin Sint, Lauren Bellhouse, Chewe Luo

**Affiliations:** a United Nations Children’s Fund (UNICEF), New York, NY, USA

## Abstract

Countries where adult male circumcision has reached high coverage should consider national early infant male circumcision (EIMC) programs where EIMC is feasible and culturally acceptable. Ministries of health that intend to set up a routine offer of EIMC should put systems in place to ensure that its introduction (1) does not compromise adult male circumcision programs, (2) does not weaken routine service delivery platforms, (3) is done safely, and (4) adheres to the rights of the child.

## BACKGROUND

Male circumcision involves the complete removal of the foreskin of the penis, the innermost layer of which is highly susceptible to HIV transmission.^1^ According to the World Health Organization (WHO), in 2006 about 30% of males globally (665 million infants, adolescents, and adults) had been circumcised.^2^ Male circumcision has historically been performed in infancy, adolescence, and adulthood in various regions of Africa for religious and cultural reasons or for ethnic identity.^2^ Circumcision is mainly performed using traditional methods, either at birth or to initiate males into adulthood at puberty. Male circumcision also has medical benefits, including the prevention of penile cancer, reduction in the transmission of some sexually transmitted infections (STIs), including HIV, and reduction in vaginal infections and cancer of the cervix in female sex partners.^3^^,^^4^

Scientific evidence and program data show that male circumcision prevents female-to-male sexual transmission of HIV.^5^ Three randomized controlled trials conducted by trained health professionals in properly equipped settings concluded that adult male circumcision reduces transmission of HIV from women to circumcised men by up to 60%.^5^^-^^9^ Based on these results, WHO and the Joint United Nations Programme on HIV/AIDS (UNAIDS) recommended in 2007 that voluntary medical male circumcision (VMMC) be made available in countries with a high HIV prevalence, generalized heterosexual HIV epidemics, and low levels of male circumcision. Fourteen countries in East and Southern Africa were prioritized for VMMC scale-up as an HIV prevention strategy: Botswana, Ethiopia, Kenya, Lesotho, Malawi, Mozambique, Namibia, Rwanda, South Africa, Swaziland, Uganda, the United Republic of Tanzania, Zambia, and Zimbabwe.^10^

Programmatically, VMMC can serve adults (males ages 15 years and older), young adolescents (boys ages 10 to 14 years), and young infants (boys ages 0 to 60 days). Njeuhmeli and colleagues estimated that scaling up VMMC in the 14 priority countries to reach 80% coverage among males ages 15 to 49 years could avert 3.4 million new HIV infections within 15 years. To achieve this goal, countries should first focus on the population in which VMMC is likely to have the most immediate and greatest impact: males ages 10 to 49 years old.^11^ This is the catch-up phase to reach the majority of males who may be currently sexually active. The sustainability phase that early infant male circumcision (EIMC) services offer can be introduced slowly to achieve coverage for 80% of males between infancy and 49 years.^12^ It is also possible to have a mixed sustainability phase of circumcision for young infants as well as young adolescents, an option that should be explored by stakeholders when exploring the introduction of this second phase of VMMC.^12^^,^^13^

Scaling up VMMC in the 14 priority countries to reach 80% coverage has the potential to avert 3.4 million new HIV infections within 15 years.

## EARLY INFANT MALE CIRCUMCISION

EIMC is medical male circumcision performed on healthy neonates from 12 to 24 hours after birth, and on young infants up to 60 days of age.^2^ It has been noted that the procedure costs less per circumcision and the process itself is much simpler in infants than in older males.^3^ There is also less risk of complication, faster wound healing, and no loss of time from work or school, factors that have been identified as barriers to acceptability of adult VMMC.^14^ Compared with adult VMMC, EIMC has also resulted in fewer surgical and postoperative adverse events.^15^^-^^17^ It also carries additional child health benefits, including reduced urinary tract infections, especially in the first 6 months of life,^18^ paraphimosis, and phimosis.

Although the sustainability phase of circumcision includes providing the services to neonates as well as young adolescents, we only look at issues related to EIMC in this article. We discuss several issues with regard to providing routine EIMC services within the public sector, as a means to sustain the gains made by adult VMMC programs. The issues derive from global meetings, United Nations expert consultations, country assessments, and policy and strategic guidance notes.

### Keys to a Successful EIMC Program

It is essential that the introduction of the routine offer of EIMC (1) does not compromise adult VMMC programs, (2) does not weaken routine service delivery platforms, (3) is done safely, and (4) adheres to the rights of the child.^19^^,^^20^ While continuing to provide adult VMMC programs, countries with high HIV and STI prevalence and high coverage of adult male circumcision programs (i.e., at least 80% of the adult male population) should consider a national EIMC program where feasible and culturally acceptable.^3^

### EIMC as a Sustainable Complement to Adult VMMC

The introduction of EIMC should not necessitate a priority shift away from adult VMMC and should not compete with other health services for children. EIMC can lead to both sustainable prevention programming as well as cost-beneficial impact over the long term.^21^

With any public health program, the key for sustainability is the commitment and investment of national authorities. Donor-funded programs and services face challenges at the end of the funding period and are often hard to sustain nationwide. The uptake of VMMC in sub-Saharan Africa emanates from commitment by ministries of health along with key stakeholders and growing acceptance in communities.^22^ With effective leadership at the national level and support from partners and implementing agencies, the same can be done with EIMC. Lessons learned from introducing EIMC services can also be used to expand the knowledge base around pediatric, biomedical HIV-prevention interventions and influence policies and decision making through operations and implementation research.

### Integration Within Routine Service Delivery Platforms

One of the key considerations for introducing EIMC is integration within routine health services. Most ongoing adult VMMC programs are donor-funded and vertical, providing only male circumcision. Within the broader context of public health and with the interest of sustaining the impact of VMMC on HIV prevention, this should not be the case for EIMC programming. The population benefiting from EIMC is male infants, so a logical platform for EIMC services would be routine services for mothers and their children, such as maternal, newborn, and child health (MNCH) services. The advantages of linking EIMC with other maternal and infant services are multiple: it would enable access to the infant at various times, such as after delivery and before discharge where appropriate, at growth monitoring, during immunization, and during child health days. However, careful consideration must be paid to avoid creating the risk of competition for resources and clients between EIMC and MNCH programs. In addition, stakeholders implementing EIMC programs should coordinate with and bolster established adult VMMC programs, ensuring that EIMC is a complementary intervention and not a diversion of resources. Successful integration of EIMC into routine health services will require strengthened relationships between maternal and child health platforms as well as with HIV programs at all levels of service delivery, and at national and international levels.

Linking EIMC with other maternal and infant services would enable access to the infant at various times, such as after delivery and before discharge, at growth monitoring, during immunization, and during child health days.

Important considerations for program investments that strengthen service delivery platforms include infrastructure support, including for surgical procedures; training, supervision, and mentoring of providers; supply chain management; and human resource investments that facilitate service delivery, such as task shifting.^23^ A systematic review in 2012 found that task shifting of adult VMMC to trained non-physicians does not increase the frequency of adverse events if performed in a supportive environment.^24^ Research is needed, and currently ongoing, to determine if this is also true for EIMC services. The task-shifting approach could have several benefits, including facilitating access to EIMCs since nonphysicians are often the main health care provider at the primary health care level, as well as minimizing costs, as demonstrated by a cost analysis of EIMCs performed by doctors compared with nurse-midwives in Zimbabwe.^25^ In addition, integration of continuous quality improvement processes into routine programming will be essential to achieve the full impact of the services.^26^^,^^27^ Research on acceptability of EIMC in various locations in East and Southern Africa has shown that fathers are often the final decision makers on when and if their sons should be circumcised.^28^^,^^29^ Providing EIMC within routine MNCH services could therefore engage fathers in a stronger way in their sons’ health.

### Quality and Safety Considerations

As with adult VMMC, the introduction and expansion of EIMC requires measures for the procedure to be carried out safely, with informed consent, and without discrimination. WHO, in collaboration with Jhpiego, has produced a manual on providing EIMC under local anesthesia to help providers and program managers deliver high-quality and safe infant male circumcision services for HIV prevention and other health benefits.^1^ This manual complements the WHO manual for adolescent and adult VMMC^30^ and focuses on the considerations and clinical best practices of circumcision for male infants under 60 days of age.

Neonatal and infant mortality rates are high in many of the male circumcision focus countries; for example, the infant mortality rate ranges from 33 infant deaths per 1,000 live births in South Africa to 73 deaths per 1,000 live births in Lesotho.^31^ Adding a routine surgical intervention to MNCH services in countries with high neonatal mortality may raise concerns. A systematic review from 2010 found few reported severe complications, but mild or moderate complications have been seen. Child circumcision tended to be associated with more complications than circumcision of neonates and infants; more complications were also associated with circumcisions performed by inexperienced providers or in non-sterile conditions.^32^ For complications, a system of referral should be in place suited to the local setting.^1^

### Patient and Parent/Guardian Rights

According to the United Nations Convention on the Rights of the Child, social welfare institutions must make the best interests of the child the primary consideration in all actions concerning children.^33^ Accurate and age-appropriate information on the protective effects of VMMC, and the risks and benefits associated with the procedure should be accessible to everyone; and the best interest of the child should be determined by taking into account diverse health, religious, cultural, and social factors—both positive and negative.

When considering the routine offer and delivery of EIMC services, national governments should weigh the issues of parental consent, the rights of the child, the health of the child, and the benefits for the wider population.^34^ It should be noted that VMMC, and the routine offer of EIMC, is appropriate in some contexts but not all. No agency recommends medical male circumcision universally, and all denounce male circumcision carried out with unsafe methods by nonmedical professionals, which can lead to infection, disfiguration, and even death.

Policies covering the issues of consent and authorization for EIMC services must be discussed and decisions made within a nation’s legal and regulatory framework.^35^ It is critical that national authorities address informed consent in EIMC programming and incorporate procedures that ensure informed consent, as should be done for all child health services. This includes prohibiting coercion, providing all information needed for decision making, giving adequate time for parent or guardian consideration, offering the option of written consent, and ensuring appropriate follow-up.^36^

It is critical that national authorities acknowledge and address the issue of informed consent and incorporate procedures that ensure informed consent into EIMC programming.

Education and information on the benefits and risks of EIMC services for both providers and clients must be offered, with an emphasis on parental rights and choice. Governments and stakeholders must ensure that parents/guardians are fully informed before they provide consent. The decision for a parent to have his or her son circumcised is a personal one, and parents/guardians should make the decision after carefully weighing the health status of the neonate and the risks and benefits, as well as religious, cultural, and personal preferences.

Information and educational materials provided during routine antenatal or child health visits should be tailored to the concerns of the caregivers and the community.^36^ This may require additional research before full EIMC service provision, including situational analysis of the target audience and testing the educational messages.^37^ EIMC may not be feasible in some settings, for example, where adolescent and adult circumcision is preferred for cultural reasons. Education and counseling will avoid reduced attendance at MNCH services due to fear of adverse events of EIMC, coercion, or not having the information to make informed decisions.

## CONCLUSION

VMMC is an effective method to prevent heterosexual transmission of HIV, and can be provided to adults, young adolescents, and young infants. Scale-up of adult VMMC in the 14 priority countries remains the focus; introduction of EIMC as a sustainability phase is recommended once adult programs have reached high coverage. EIMC programs are not replications of adult and adolescent services, but require thoughtful consideration of many infant-specific issues. EIMC service provision should be context-specific, and led by national authorities with support from implementing agencies.

EIMC services should be offered as part of routine MNCH services and used as an opportunity to strengthen newborn and child health services overall. The quality and safety of the services remain paramount: the rights of the child should be protected at all times by providing complete information to parents and guardians to inform their decisions, giving them adequate time to consider their options, offering the option of written consent, and ensuring appropriate follow-up.

Although the impact of EIMC on HIV incidence and overall prevalence will not be realized immediately, the routine offering of EIMC services will provide longer-term impact, benefiting the health of the child as well as protecting against heterosexual transmission of HIV at both the individual and population level. Performing medical male circumcision in infancy will provide lifelong benefits for the child and contribute to sustaining the gains made from adult VMMC programs.

## Notes

**Peer Reviewed**
